# Purification of Phenolic Compounds from *Camellia polyodonta* Flower: Composition Analysis, Antioxidant Property, and Hypolipidemic Activity In Vitro and In Vivo

**DOI:** 10.3390/antiox13060662

**Published:** 2024-05-28

**Authors:** Zhuoya Xiang, Li Liu, Zhou Xu, Qingbo Kong, Heng Liang, Shiling Feng, Tao Chen, Lijun Zhou, Hongyu Yang, Chunbang Ding

**Affiliations:** 1 College of Life Science, Sichuan Agricultural University, Ya’an 625014, China; xiangzhuoya2015@163.com (Z.X.);; 2 Institute of Agro-Products Processing Science and Technology (Institute of Food Nutrition and Health), Sichuan Academy of Agricultural Sciences, 60 Shizishan Road, Chengdu 610066, China; 3 Panxi Crops Research and Utilization Key Laboratory of Sichuan Province, Xichang University, Xichang 615000, China

**Keywords:** *Camellia polyodonta* flower, phenolic compounds, purification, antioxidant activity, hypolipidemic activity

## Abstract

*Camellia polyodonta* flowers are rich sources of phenolics and less attention has been paid to their potential biological activity. This study aims to explore the crude extracts and resulting purified fractions (CPFP-I, II, III, and IV) through compositional analysis and antioxidant and hypolipidemic activities in vitro and in vivo. Among four fractions, CPFP-II contained the highest total phenolic content and flavonoid content, while CPFP-III exhibited the greatest total proanthocyanidin content. Among the 14 phenolic compounds, CPFP-II displayed the highest content of procyanidin B2, B4, and C1, whereas CPFP-III contained the highest amount of 1,2,3,6-tetragalloylglucose. The DPPH, ABTS, and FRAP assessments demonstrated a consistent trend: CPFP-II > CPFP-III > CPFP-I > CPFP-IV. In vivo experiments showed that that all four fractions significantly reduced lipid levels in hyperlipidemic *C. elegans* (*p* < 0.05), with CPFP-II exhibiting the most potent effect. Furthermore, CPFP-II effectively bound to bile acids and inhibited the enzymatic activity of pancreatic lipase in vitro. Consequently, CPFP-II should be prioritized as a promising fraction for further exploration and should provide substantial support for the feasibility of the *C. polyodonta* flower as a natural alternative.

## 1. Introduction

With the rapid development of society, the availability of food has become abundant. Consequently, the spectrum of diseases among residents has shifted from ailments caused by hunger and nutrient deficiencies to chronic metabolic conditions like diabetes and hyperlipidemia resulting from excessive nutrition and poor dietary choices [1]. Of these conditions, hyperlipidemia has emerged as a significant health concern in recent years. This disorder pertains to lipid metabolism and is characterized by elevated levels of total serum cholesterol (TC), triglycerides (TGs), or low-density lipoproteins (LDLs) [2]. Addressing these metabolic diseases related to food consumption necessitates an emphasis on the quantity and composition of the food supply. In the initial stages of treatment, lipid-lowering drugs such as statins, ezetimibe, and resins were employed. However, these medications come with drawbacks, like a single-target approach, a high risk of liver and kidney damage, and the potential for rapid rebound effects [3]. Consequently, there has been a substantial focus on researching new natural bioactive compounds derived from herbs and edible materials for the treatment of hyperlipidemia. Notably, flavonoids [4], polyphenols [5], saponins [6], and polysaccharides [7] have been extensively studied. These bioactive components have been shown to play a role in combating oxidative stress in hypertension treatment [8,9]. Additionally, their potential in reducing lipid levels is likely linked to the inhibition of key enzymes, such as pancreatic lipase [10].

*Camellia polyodonta* is a distinctive edible oil plant, widely distributed in the high mountains and hilly districts of subtropical regions in southern China [11]. Its flowers bloom during the winter, exhibiting a prolonged blooming period, and are highly ornamental. In traditional Chinese herbal medicine, these flowers were utilized to staunch bleeding and treat burns. Modern research has also highlighted the remarkable potential of bioactive compounds found in its seeds and shells, showcasing antioxidant, anti-inflammatory, anticancer, and hypolipidemic properties [12,13,14]. Nonetheless, the attention directed toward the flower itself has been limited. Our prior research, however, has unveiled that *C. polyodonta* flowers contain elevated levels of phenolic compounds, encompassing flavonoids, proanthocyanidins, and tannins [15]. Furthermore, proanthocyanidins and tannins have been proven to possess significant biological activities, including antioxidant and hypolipidemic effects [16]. These compounds are considered the most abundant phenolics within *C. polyodonta* flowers. Consequently, there is a pressing need for further enrichment of phenolic compounds to enhance the potential of *C. polyodonta* flower extracts as effective future functional food ingredients.

The potential for *C. polyodonta* flowers to serve as promising functional food ingredients is reinforced by our previous findings, which underscored their notably higher phenolic compound content compared to other species and certain functional foods [15]. However, no reports have delved into the potential biological activities of distinct enriched materials derived from *C. polyodonta* flowers. This study marks the first instance of using AB-8 resins for the purification of hyperlipidemia-related compounds from *C. polyodonta* flowers. This study also encompasses compositional analysis, in vitro antioxidant activity assessments, and both in vitro and in vivo hypolipidemic activity evaluations of varied concentrated phenolic productions. These efforts are aimed at establishing a foundation for the future development of functional foods or drugs sourced from *Camellia polyodonta* flowers.

## 2. Materials and Methods

### 2.1. Materials

The flowers of *C. polyodonta* were collected from Zhougong Mountain (Ya’an, China) in December 2021. The entire flowers were freeze-dried immediately. Subsequently, they were ground into 60-mesh particles, sealed, and stored at −20 °C for testing purposes.

### 2.2. Chemicals and Reagents

Folin–Ciocalteu phenol reagent, Na_2_CO_3_, formic acid, Al(NO_3_)_3_, NaNO_2_, HCl, H_2_SO_4_, methanol, ethanol, and Trolox were obtained from Chengdu Kelong Chemical Reagent Works (Chengdu, China). 1,1-Diphenyl-2-picrylhydrazyl (DPPH, >99.7%), 2,2′-azino-bis(3-ethylbenzo thiazoline-6-sulfonic acid) diammonium salt (ABTS+, >99.7%), Oil red O (ORO), p-ntrophenyl laurate (*p*NP laurate), and chromatographic-grade methanol and acetonitrile were purchased from American Sigma (St. Louis, MO, USA). Standard compounds, including rutin, afzelin, astragalin, kaempferol-3-*O*-rutinoside, isoquercitrin, (+)-catechin, procyanidin B2, cyanidin-3-*O*-glucoside, 1,2,3,6-tetragalloylglucose, simvastatin, orlistat, glycocholic acid, cholic acid, taurocholic acid, porcine pancreatic lipase, and tris-HCl buffer solution, were purchased from Shanghai Yuanye Bio-Technology Co., Ltd. (Shanghai, China). Standard compounds, including procyanidin B1, procyanidin B4, and procyanidin C1, were purchased from Chengdu Biopurify Phytochemicals Ltd. (Chengdu, China).

### 2.3. Preparation of Crude Camellia polyodonta Flower Extract

According to our previous study [15], a total of 100 g of dry *Camellia polyodonta* flower powder was subjected to extraction using 1000 mL of 70% (*v*/*v*) methanol, with ultrasound assistance for 30 min at a temperature of 40 °C. After this, the resulting supernatant was obtained via centrifugation (6654× *g*, 10 min), and this extraction process was repeated two times. Subsequently, the supernatant was concentrated using Hei-VAP Advantage rotary evaporators from HEIDOLPH (Schwabach, Germany), employing reduced pressure at 45 °C. The concentrated solution was then freeze-dried. The final product, the *Camellia polyodonta* flower extract, was carefully stored in a drying cabinet, shielded from light, for its intended future applications.

### 2.4. Pretreatment of Macroporous Resins

The AB-8 resins underwent a series of pretreatment steps. Initially, they were soaked in 95% ethanol for a duration of 24 h. After the ethanol was removed, the resins were thoroughly washed with distilled water until any discernible ethanol odor was eliminated. Subsequently, the resins were immersed in a 4% NaOH solution for a period of 2 h. Following this step, the resins were washed twice with distilled water until they reached a neutral state. The resins, having undergone the aforementioned treatments, were then soaked in a 1 mol/L HCl solution for a period of 2 h. Finally, the resins were once again washed meticulously with distilled water until they were neutral. This washing process was repeated a second time. The resins that had undergone these thorough pretreatment procedures were stored in distilled water and reserved for subsequent use.

### 2.5. Enrichment of Phenolics by Resin Column

Enrichment experiments were conducted using four glass columns (3 cm × 60 cm), which were packed with AB-8 resins. The bed volumes of the wet-packed AB-8 resins, standardized by weight, were set at 197 mL. The crude extract of *Camellia polyodonta* flowers (CPFP) was prepared at a concentration of 9 mg/mL and dissolved in distilled water. This CPFP solution was meticulously applied to the column. Once the adsorption equilibrium was attained, the column was subjected to sequential elution using different ethanol concentrations: 800 mL of distilled water, followed by 10% (*v*/*v*) ethanol, 20% (*v*/*v*) ethanol, 30% (*v*/*v*) ethanol, 40% (*v*/*v*) ethanol, and 50% (*v*/*v*) ethanol. The elution was carried out at a constant flow rate of 0.5 mL/min and a temperature of 24 °C.

The resulting eluates, referred to as CPFP-I, CPFP-II, CPFP-III, CPFP-IV, and CPFP-V, were collected individually. Subsequently, each fraction underwent a process of ethanol removal using Hei-VAP Advantage rotary evaporators from HEIDOLPH (Schwabach, Germany), operating under reduced pressure at 50 °C. Following this, every fraction was subjected to freeze-drying. The dried fractions were weighed, stored at room temperature in a drying cabinet, and reserved for subsequent use.

### 2.6. Determination of Total Phenolic Content

The determination of the total phenolic content (TPC) followed the previous method [15], albeit with some adaptations. For each elution fraction sample, an appropriate dilution was prepared. Specifically, 20 μL of the sample solution was mixed with 20 μL of Folin–Ciocalteu reagent and allowed to react for a duration of 5 min. Subsequently, 160 μL of a 5% Na_2_CO_3_ solution was added to the mixture, which was then left to react for 60 min. The resulting mixture’s absorbance was measured at 765 nm. The calibration curve was established using gallic acid, resulting in the equation *y* = 0.0078*x* − 0.0017 (*R*^2^ = 0.9996). The linear range of this curve was determined to be 2.32–148.75 μg/mL. The outcomes of the analysis were reported as milligrams of gallic acid equivalent (GAE) per gram of dry weight (DW).

### 2.7. Determination of Total Flavonoid Content

The total flavonoid content (TFC) was assessed using the aluminum chloride colorimetric method. Each elution fraction sample was appropriately diluted. In this procedure, 15 μL of a 5% (m/v) NaNO_2_ solution was mixed with 20 μL of the sample, and the mixture was allowed to react for 6 min at room temperature. Subsequently, 10 μL of a 10% Al(NO_3_)_3_ solution was added and left to react for 5 more min. Following this, 30 μL of a 1 mol/L NaOH solution was introduced, and the resulting mixture’s absorbance was measured at 510 nm. The calibration curve was generated using catechin as the reference compound, yielding the equation *y* = 0.0017*x* + 0.0472 (*R*^2^ = 0.9967), with a linear range spanning from 7.81 to 1000 μg/mL. The results were quantified and reported in terms of milligrams of catechin equivalent (CE) per gram of dry weight (DW).

### 2.8. Determination of Total Proanthocyanidin Content

The proanthocyanidin content (TPAC) was quantified using the vanillin assay. For each elution fraction sample, an appropriate dilution was prepared. In this process, 20 μL of the sample was combined with 100 μL of a 1% (m/v) vanillin solution in methanol, followed by the addition of 100 μL of 4% (*v*/*v*) HCl in methanol. The resultant mixture was incubated at 37 °C for 20 min and subsequently measured at 500 nm. To establish the calibration curve, procyanidin B2 was utilized as the reference compound, yielding the equation *y* = 0.4367*x* + 0.0026 (*R*^2^ = 0.9996), with a linear range spanning from 30 to 1000 μg/mL. The outcomes of the analysis were reported as milligrams of procyanidin B2 equivalent per gram of dry weight (DW).

### 2.9. Determination of Antioxidant Activity

The antioxidant activity of different purified fractions was evaluated using two radicals (DPPH• and ABTS^•+^) and the ferric-reducing ability (FRAP) assay.

To assess the radical scavenging effect on DPPH•, specifically, 100 µL of the sample was combined with 100 µL of a 0.2 M DPPH solution. The mixture was then incubated in the dark for a duration of 30 min before the absorbance was measured at 517 nm.

For the ABTS^•+^ radical cation assessment, a mixture of 160 μL of ABTS^•+^ solution and 40 μL of the sample was prepared promptly. This mixture was left at room temperature in the dark for 6 min, after which the absorbance at 734 nm was measured. Distilled water was employed as a blank, and vitamin E (VE) served as the positive control. The inhibitory concentration at 50% (IC_50_) reflected the antioxidant concentration of the tested samples needed to neutralize 50% of the initial concentration of free radicals.

The FRAP assay involved adding 30 μL of the sample to 256 μL of the FRAP reagent. This mixture was kept at room temperature for 30 min before the absorbance was measured at 593 nm.

### 2.10. Compositional Analysis of Purified Fraction

The Agilent LC-1290 HPLC system (Agilent, Santa Clara, CA, USA) was employed to analyze the purified CPFP fractions. These fractions, measured at 5 μL each, were separated using an Infinity Lab Poroshell 120 PFP column (4.6 × 100 mm, 2.7 µm). The separation process involved a mobile phase comprising solvent A (0.1% formic acid in water) and solvent B (acetonitrile). The elution steps adhered to the methodology outlined in a prior study [15]: 0–10 min with a gradient from 5% to 10% B; 10–20 min with a gradient from 10% to 20% B; 20–35 min with a gradient from 20% to 40% B; 35–36 min with a gradient from 40% to 45% B; and 36–37 min with a gradient from 45% to 90% B. The total flow rate during this process was maintained at 0.8 mL/min, and detection was carried out at wavelengths of 280 and 350 nm. For quantification, fourteen phenolic compounds were assessed, and corresponding standard compounds were used for calibration. The quantified results were expressed in micrograms per gram of dry weight (µg/g DW).

### 2.11. Evaluation of In Vitro Hypolipidemic Activity

#### 2.11.1. Bile-Acid-Binding Capacity

The bile-acid-binding capacity assessment followed the procedure of [17]. In brief, different concentrations of elution fractions underwent a simulated gastric environment involving treatment with pepsin (10 mg/mL) and HCl solution (0.01 mol/L) at 37 °C with continuous agitation for 1 h followed by adjustment in pH to 6.3 using 0.1 mol/L NaOH and subsequent exposure to porcine pancrezyme solution (10 mg/mL) at 37 °C, with continuous agitation for an additional hour to mimic intestinal conditions. Afterward, the solution was mixed with 2 mL of 0.3 mmol/L sodium cholate, sodium glycocholate, or sodium taurocholate, respectively, and incubated at 37 °C for 1 h with continuous agitation. Centrifugation (6654× *g*, 5 min) separated the supernatant for bile acid determination. Simvastatin (31.25 μg/mL) served as the positive control. Unbound bile acid concentration was gauged using a standard curve prepared with sodium cholate, sodium glycocholate, and sodium taurocholate. The binding capacity (%) was computed as (C_1_ − C_2_)/C_1_ × 100, with C_1_ signifying the initial bile acid concentration and C_2_ representing the unbound bile acid concentration. This result was compared against a 0.1 mol/L phosphate buffer (pH 6.3), with each sample tested in duplicate against each bile acid.

#### 2.11.2. Pancreatic Lipase Inhibitory Effect

The assessment of the inhibitory effect on pancreatic lipase activity followed the method of [10] with minor adjustments. Various concentrations of CPFP fractions (50 μL) were individually mixed with pancreatic lipase (5 mg/mL, 200 μL) and incubated for 15 min at 37 °C. Following this, the substrate p-nitrophenyl laurate (pNP laurate, 3 mg/mL, 50 μL) was introduced, and the mixture was incubated for an additional 45 min at 37 °C. Subsequently, the absorbance of this solution was measured at 405 nm. Orlistat was utilized as the positive control. The inhibitory effect was analyzed using the following equation:

Inhibitory effect (%) = (1 − (*A*_1_ − *A*_2_)/(*A*_3_ − *A*_4_)) × 100,

where *A*_1_ is the absorbance obtained under conditions with the sample; *A*_2_ is the absorbance of the sample blank control without lipase; *A*_3_ is the absorbance of the reaction solution without the sample, and *A*_4_ is the absorbance of the blank control without the sample and lipase.

### 2.12. Hypolipidemic Activity In Vivo

#### 2.12.1. Caenorhabditis Elegans Strains and Maintenance

The hypolipidemic activity of the four CPFP fractions was evaluated in vivo using the hyperlipidemia *C. elegans* model induced by 5 mM glucose (Supplementary Material Figure S1). The wild-type N2 worms were cultured on nematode growth media (NGM) plates with *E. coli* OP50 bacteria, maintained at 20 °C. The worms were synchronized and subsequently exposed to the four CPFP fractions (250, 500, and 1000 μg/mL, final concentrations), which were mixed with *E. coli* OP50 on NGM plates.

#### 2.12.2. Oil Red O (ORO) Staining and Triglyceride (TG) Content

For Oil Red O (ORO) staining, synchronized worms were subjected to treatment with the four CPFP fractions. Following a 60 h exposure period, the worms were washed three times with M9 buffer and subsequently fixed and dehydrated using 60% isopropanol for a duration of 30 min. The next step involved staining the worms with ORO stocking solution (5 mg/mL) for a period of 6 h at room temperature in darkness. After this staining period, the worms were washed three times with an M9 buffer and then mounted for imaging under a fluorescence microscope (Nikon DS-Ril, Nikon Corporation, Tokyo, Japan).

For the determination of triglyceride (TG) levels, each group consisting of over 1000 worms was washed three times with the M9 buffer after being removed from the NGM plates. Subsequently, ultrasonic disruption was employed to homogenize the worms and release triglycerides, and the resulting homogenate underwent centrifugation at 936× *g* for 10 min to collect the supernatant. The TG content was normalized by the protein content, which was measured using an available kit (Nanjing Jiancheng Bioengineering Institute, Nanjing, China).

## 3. Results and Discussion

### 3.1. Total Phenolic, Flavonoid, and Proanthocyanidin Contents

Phenolic compounds were identified as the crucial constituents within the ethanol extract of *C. polyodonta* flower, prompting the necessity to obtain a phenolic-rich fraction for deeper mechanistic exploration. For this purpose, AB-8 macroporous resin was utilized for the preliminary separation of phenolic compounds, seeking to ascertain whether the phenolic-rich fraction was the primary target constituent. This led to the application of varying concentrations of ethanol/water solvents in the subsequent purification process to secure a phenolic fraction of heightened purity. This endeavor resulted in five distinct fractions: Fraction I (CPFP-I), acquired using 10% aqueous ethanol, Fraction II (CPFP-II), with 20% aqueous ethanol, Fraction III (CPFP-III), with 30% aqueous ethanol, Fraction IV (CPFP-IV), with 40% aqueous ethanol, and Fraction V (CPFP-V), with 50% aqueous ethanol.

As indicated in Table 1, among these five fractions, CPFP-II exhibited the highest eluting yield, accounting for 46.27% of the total yield, followed by notable decreases in CPFP-III, CPFP-I, CPFP-IV, and CPFP-V (31.37%, 7.83%, 7.61%, and 6.90%, respectively). The total phenolic content, flavonoid content, and proanthocyanidins content of CPFP-I, CPFP-II, and CPFP-III were all notably higher than those of CPFP, whereas those of CPFP-IV were slightly superior to those of CPFP. This observation indicated significant enrichment of bioactive components in these four fractions. CPFP-II exhibited the highest levels of total phenolic content (909.27 mg GAE/g DW) and total flavonoid content (658.36 mg CE/g DW), while CPFP-III had the highest total proanthocyanidin content (175.56 mg PB2E/g DW). This is obviously higher than in the study by Chen et al. [18], who detected the 30 species of flowers with TPC to be in the range of 8.44–97.05 mg GAE/g DW and with TFC to be in the range of 4.23–90.51 mg RE/g DW. Notably, the CPFP-II and CPFP-III fractions contained over 48% and 30% of the total phenolics, respectively. The majority of phenolics absorbed by the AB-8 resins were effectively obtained when employing 20% and 30% ethanol solvents for elution. The polarity of the eluting solvent wielded a substantial influence on desorption capacity, with more polar solvents displaying a higher desorption selectivity for polyphenols, possibly attributed to the presence of polar phenolic hydroxyl groups and explained by the principle of “similarity and intermiscibility” [19]. This observation also suggested the prevalence of highly polar polyphenols within the *C. polyodonta* flower. Overall, considering the considerable biological activities exhibited by higher levels of bioactive components, the purified extracts enriched with substantial phenolics can be harnessed as efficacious constituents within the pharmaceutical industry. Consequently, the CPFP-I, CPFP-II, CPFP-III, and CPFP-IV purified fractions were collected for subsequent comparison of their hypolipidemic activity.

### 3.2. Quantification of Phenolic Compounds

**Figure 1:**
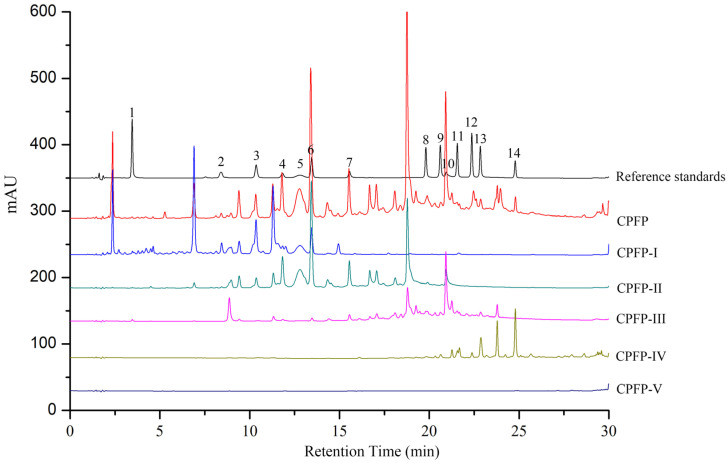
HPLC chromatograms of fourteen standards of CPFP and its different purified fractions (CPFP-I, CPFP-II, CPFP-III, CPFP-IV, and CPFP-V) at 280 nm wavelength; 1, gallic acid; 2, procyanidin B1; 3, (+)-catechin; 4, procyanidin B2; 5, procyanidin B4; 6, epicatechin; 7, procyanidin C1; 8, rutin; 9, isoquercitrin; 10, 1,2,3,6-tetragalloylglucose; 11, kaempferol-3-*O*-rutinoside; 12, astragaline; 13, quercitrin; 14, afzelin.

To investigate the composition of CPFP and its five fractions, high-performance liquid chromatography (HPLC) was employed. As depicted in Figure 1, CPFP displayed a substantial presence of polyphenolic constituents, detectable within the 10–25 min retention time range. After the fractionation of CPFP using macroporous resins, CPFP-II emerged as the fraction with the highest enrichment of constituents, although its chromatographic profile differed noticeably from the others. Notably, the bioactive constituents in CPFP-I were primarily eluted from 1 to 15 min, while CPFP-III predominantly contained bioactive compounds in the range of 18–22 min and CPFP-IV from 22 to 25 min. CPFP-V, on the other hand, exhibited no constituents, signifying the comprehensive enrichment of CPFP-I, CPFP-II, CPFP-III, and CPFP-IV for bioactive components derived from CPFP.

Furthermore, the quantification of the 14 phenolic compounds is presented in Table 2, encompassing one phenolic acid (gallic acid), four procyanidins (procyanidin B1, procyanidin B2, procyanidin B4, and procyanidin C1), two flavanols ((+)-catechin and (-)-epicatechin), six flavonols (rutin, afzelin, astragalin, kaempferol-3-*O*-rutinoside, quercitrin, and isoquercitrin), and one ellagitannin (1, 2, 3, 6-tetragalloylglucose). Notably, CPFP-II exhibited the highest enrichment of procyanidins (B2, B4, C1) and epicatechin among all the fractions, surpassing the levels in the other two fractions by significant margins. The content of procyanidins B2 in CPFP-II was 4.56 times that of CPFP-I and 14.33 times that of CPFP-III. Similarly, the contents of procyanidins B4 and procyanidins C1 in CPFP-II were 2.04 and 21.10 times that of CPFP-I, respectively, and 3.58 times that of CPFP-III in the case of procyanidins C1. The content of epicatechin in CPFP-II was 3.81 times that of CPFP-I and 35.11 times that of CPFP-III. Notably, procyanidins are recognized for their lipid-lowering properties. For example, Lu et al. [20] demonstrated that proanthocyanidins effectively reduced elevated triglyceride (TG) and high-density lipoprotein (HDL) levels induced by a high-fat diet in grass carp. CPFP-III exhibited the highest content of 1,2,3,6-tetragalloylglucose (43.69 mg/g DW), known for its significant inhibitory effect on lipid peroxidation, as demonstrated by a previous study [21]. Furthermore, CPFP-IV was the most enriched in flavonols (kaempferol-3-*O*-rutinoside, quercitrin, and isoquercitrin). Many of these phenolic compounds in CPFP-II and CPFP-III have been reported to possess various biological and pharmacological effects, including hypolipidemic and anticancer properties, as well as antioxidant activities [11].

### 3.3. Antioxidant Activity

The extracts of the *C. polyodonta* flower are rich in polyphenols, which are known for their potent antioxidant properties. To comprehensively assess the antioxidant potential of CPFP and its five fractions, DPPH, ABTS, and FRAP assays were employed, collectively evaluating their free radical scavenging capabilities through distinct mechanisms. The DPPH and ABTS assays have been widely utilized to gauge the free radical scavenging abilities of extracts, including DPPH• and ABTS^•+^, providing insights into their hydrogen-donating and chain-breaking capacities [22]. On the other hand, the FRAP assay quantifies the antioxidants’ capability to reduce Fe^3+^ to Fe^2+^ in the presence of TPTZ. This reduction power corresponds to the substances’ electron-donating capacity, with higher absorbance values indicating stronger reducing power [23].

**Figure 2:**
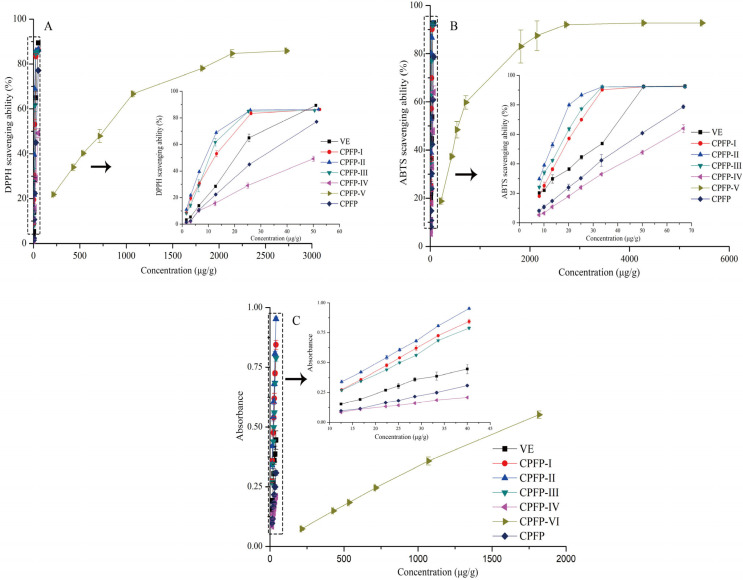
The DPPH (**A**), ABTS (**B**) and radical scavenging values and ferric reducing power (FRAP, **C**) of VE, CPFP, and its different purified fractions (CPFP-I, CPFP-II, CPFP-III, and CPFP-IV). The arrow (→) points to an enlargement of the contents of the dashed box.

As depicted in Figure 2, the DPPH• and ABTS^•^+ radical scavenging activities of all six CPFP fractions were apparent and demonstrated a positive correlation with increasing concentrations. To determine the effectiveness of radical scavenging, IC_50_ values for DPPH and ABTS assays were calculated for the various CPFP samples. Notably, CPFP-II extract displayed the most robust DPPH• and ABTS^•^+ radical scavenging activity, with IC_50_ values of 8.25 ± 0.10 μg/mL and 11.93 ± 0.07 μg/mL, respectively. These values surpassed those of VE (17.86 μg/mL and 6.08 μg/mL), signifying its remarkable antioxidant potency. The trend of DPPH• and ABTS^•^+ scavenging activity followed this order: CPFP-II > CPFP-III > CPFP-I > VE > CPFP > CPFP-IV > CPFP-V. This suggests that CPFP-II may have more effectively enriched bioactive compounds compared to CPFP-III, CPFP-I, CPFP-IV, and CPFP-V. These fractions were obtained through AB-8 macroporous resins using different polar solvents, leading to variations in their compositions and content. However, the radical scavenging activity of phenolic compounds relies on the positioning and number of hydroxyl groups relative to the carboxyl functional group [24]. Therefore, the elevated antioxidant activity of CPFP-II might be attributed to its unique composition and chemical structure, including a higher content of proanthocyanidins as outlined in Table 2. Additionally, a strong correlation was observed between the IC_50_ of DPPH and ABTS with procyanidin B1 (*R* = 1, *p* < 0.01), (+)-catechin (*R* = 1, *p* < 0.01), (-)-epicatechin (*R* = 1, *p* < 0.01), procyanidin B4 (*R* = 1, *p* < 0.01), rutin (*R* = 1, *p* < 0.01), afzelin (*R* = 1, *p* < 0.01), astragalin (*R* = 1, *p* < 0.01), kaempferol-3-*O*-rutinoside (*R* = 1, *p* < 0.01), quercitrin (*R* = 1, *p* < 0.01), isoquercitrin (*R* = 1, *p* < 0.01), and 1,2,3,6-tetragalloylglucose (*R* = 1, *p* < 0.01) by correlation analysis. Studies have reported that the free hydroxyl group in phenolic compounds is the main cause of antioxidant activity, and the greater the number of free hydroxyl groups, the stronger the antioxidant activity, which may be the reason for the strong correlation between phenolic compounds and antioxidant activity [25].

Reducing power refers to the ability of compounds, often due to the presence of hydroxyl groups, to transfer electrons, thereby serving as a measure of their antioxidant capacity. As illustrated in Figure 2, all absorbance values exhibited a linear and dose-dependent increase, indicating the reliability of the reducing power assay for determining the sample concentrations. The results of the FRAP assay mirrored the trends observed in the DPPH• and ABTS^•^+ scavenging activities, with the order being CPFP-II > CPFP-I > CPFP-III > VE > CPFP > CPFP-IV > CPFP-V. Collectively, the results of the three antioxidant assays strongly indicated the substantial antioxidant potential of CPFP-II, followed by CPFP-I and CPFP-III, while CPFP-V exhibited a significantly lower antioxidant capacity. In light of these findings, CPFP-II, CPFP-III, CPFP-IV, and CPFP-V fractions were selected for subsequent investigations comparing their hypolipidemic activities both in vitro and in vivo.

### 3.4. Hypolipidemic Activity of CPFP and Its Fractions In Vitro

#### 3.4.1. Bile-acid-Binding Capacity

**Figure 3:**
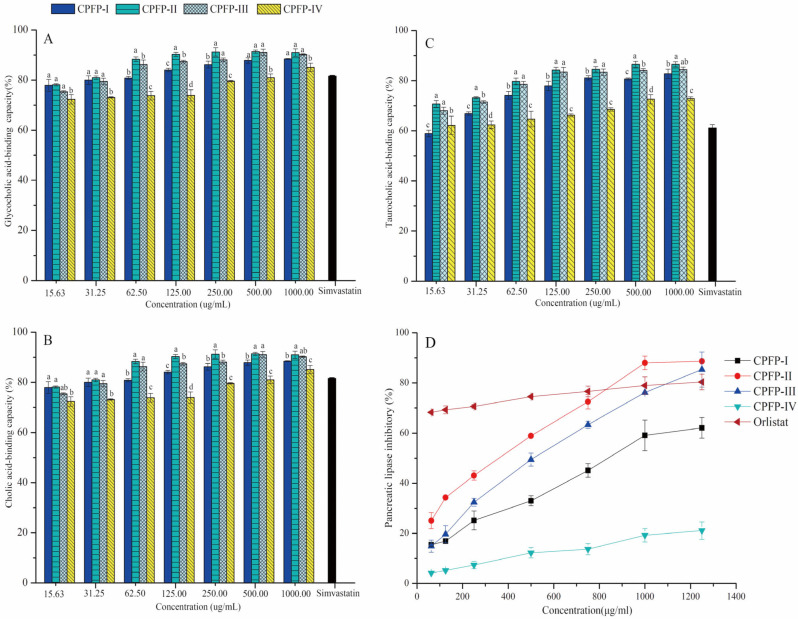
Glycocholic-acid-binding capacity (**A**), cholic-acid-binding capacity (**B**), taurocholic-acid-binding capacity (**C**), and pancreatic lipase inhibition (**D**) of CPFP-I, CPFP-II, CPFP-III, and CPFP-IV. The significant (*p* < 0.05) differences are shown by data bearing different lowercase letters (a–d) in same bar.

Natural products that bind to bile acids and enhance their fecal excretion can lead to decreased levels of plasma cholesterol [26]. The bile-acid-binding capacities of the different purified CPFP fractions are presented in Figure 3. The capacities to bind to glycocholic, cholic, and taurocholic acids varied among the four fractions, and the binding capacities were concentration-dependent and statistically significant (*p* < 0.05). Overall, CPFP-II exhibited significantly higher binding capacities and CPFP-IV exhibited significantly lower binding capacities compared to the other fractions (*p* < 0.05), except for the taurocholic-acid-binding capacity at the lowest concentration (Figure 3C).

For glycocholic-acid-binding capacities (Figure 3A), as the concentration was below 500 μg/mL, the binding capacities of all four CPFP fractions increased proportionally to the concentration. At a concentration of 500 μg/mL, CPFP-I, CPFP-II, CPFP-III, and CPFP-IV exhibited binding capacities of 87.44%, 88.77%, 88.61%, and 83.66%, respectively, reaching their highest values. Although no significant differences were observed in the cholic-acid-binding capacity between CPFP-I, CPFP-II, and CPFP-III, their capacities were notably higher than CPFP-IV and simvastatin (31.25 μg/mL). The substantial glycocholic-acid-binding capacity of CPFP-II indicated that the abundant polyphenol content contributed to a higher binding efficiency, potentially reducing the cholesterol content in the body and rapidly and significantly lowering blood lipids [27].

Cholic-acid-binding capacities of CPFP-II were significantly higher than those of CPFP-I and CPFP-IV (Figure 3B). At a concentration of 500 μg/mL, the binding capacities for CPFP-I, CPFP-II, and CPFP-III reached their peak values of 87.79%, 91.36%, and 91.04%, respectively. The binding capacities of taurocholic acids for all four CPFP fractions were lower than those for glycocholic and cholic acids (Figure 3C). As the concentration remained below 500 μg/mL, the taurocholic-acid-binding capacities of all four CPFP fractions increased in line with the concentration. At a concentration of 500 μg/mL, the binding capacities for CPFP-I, CPFP-II, CPFP-III, and CPFP-IV were 80.69%, 86.52%, 84.08%, and 72.61%, respectively.

In conclusion, the findings demonstrated that, among the four CPFP fractions, CPFP-II exhibited the highest bile-acid-binding capacity, followed by CPFP-III, suggesting that CPFP-II could be particularly effective in reducing lipid levels in vitro. This bile-acid-binding capacity can be attributed to the characteristics of polyphenols and their composition. The interaction between the carboxyl groups of bile acids and the phenolic hydroxyl groups of polyphenols is mediated through hydrophobic interactions, which, in turn, can hinder lipid absorption [28]. The results also implied that a higher polyphenol content led to an improved binding efficiency with the four cholate compounds, aligning with findings from Hamauzu and Suwannachot et al. [29]. In addition, the types of phenolic compounds also affected the bile-acid-binding capacity. CPFP-II is rich in flavan-3-ol polymerized proanthocyanidins and Hamauzu and Suwannachot et al. [29] also indicated that insoluble proanthocyanidins in persimmon extract have a strong bile-acid-binding ability.

#### 3.4.2. Inhibition of Pancreatic Lipase

Pancreatic lipase plays a crucial role in the metabolism of dietary lipids [30]. The inhibitory effects of the four CPFP fractions on pancreatic lipase activity are illustrated in Figure 3D. The inhibitory capacity of these fractions exhibited a clear dose-dependent relationship, which can be attributed to the binding of polyphenols with enzymes, potentially hindering the active site and enzymatic activity of lipase [31]. Both CPFP-II and CPFP-III displayed inhibition rates exceeding 80% of lipase activity at a concentration of 1250 μg/mL, which was obviously higher than the inhibition rate (50–80%) of pancreatic lipase of citrus and grape pomace polyphenol extract (100 mg/mL) [32]. CPFP-I exhibited an inhibition rate of approximately 60% of lipase activity at the same concentration. Conversely, CPFP-IV achieved an inhibition rate that did not surpass 50%, even at 1250 μg/mL. In comparison, the positive control, orlistat, inhibited 68% of lipase activity at 62.50 μg/mL and 80% at 1250 μg/mL, suggesting relatively weaker inhibition compared to CPFP-II and CPFP-III. The 50% maximal inhibitory concentrations (IC_50_) of CPFP-I, II, and III were determined as 841.37 ± 141.12, 247.78 ± 5.93 μg/mL, and 409.53 ± 14.47, respectively. The study revealed that CPFP-II was particularly effective in inhibiting pancreatic lipase compared to the other fractions. Additionally, a strong negative correlation was observed between the total phenolic content (TPC) and total proanthocyanidin content (TPAC), with the IC_50_ values for lipase inhibition (*R* = −0.952 and *R* = −0.844, *p* < 0.01), in line with findings by Cai et al. [33]. For phnenolic compounds, procyanidin B2 (*R* = 1, *p* < 0.01), procyanidin C1 (*R* = 1, *p* < 0.01), and (-)-epicatechin (*R* = 1, *p* < 0.01) also showed a strong correlation with the IC_50_ values for lipase inhibition. CPFP-II has a higher content of proanthocyanidins with a higher number of hydroxyl groups in its structure, which may also be the reason for its higher inhibitory ability of pancreatic lipase. Therefore, polyphenols are likely the key compounds responsible for lipase inhibition, and the inhibitory effect on lipase by polyphenols might follow a mixed-competitive mode, as previously demonstrated [34].

### 3.5. Evaluation of Hypolipidemic Efficacy In Vivo in a C. elegans Model

**Figure 4:**
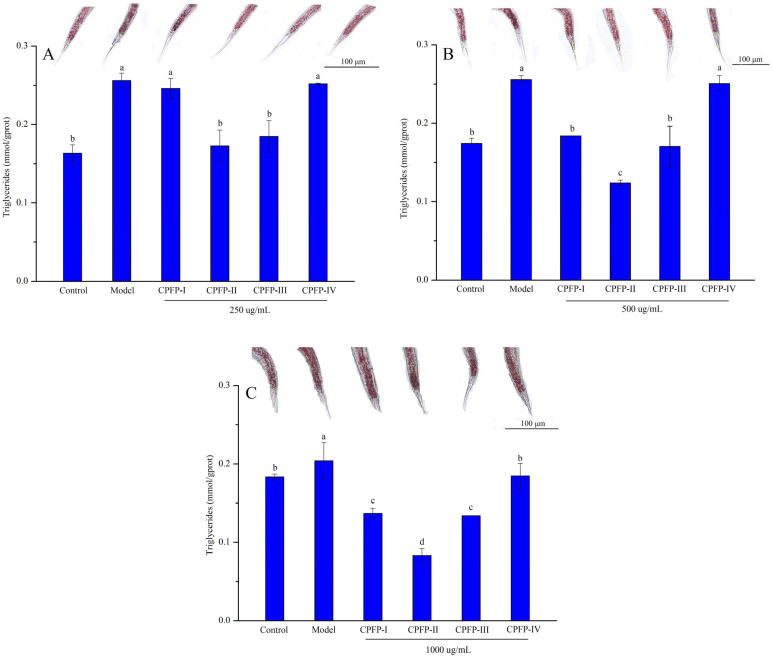
Effect of different CPFP-I, CPFP-II, CPFP-III, and CPFP-IV concentrations on ORO staining of *C. elegans* and the triglyceride (TG) content in high-fat N_2_. (**A**), 250 μg/mL; (**B**) 500 μg/mL; (**C**) 1000 μg/mL. The significant (*p* < 0.05) differences are shown by data bearing different lowercase letters (a–d) in same bar.

*C. elegans* has proven to be a valuable model organism for hypolipidemic studies due to the highly conserved nature of lipid-metabolism-related genes and signaling pathways between *C. elegans* and humans [35]. Figure 4 presents the investigation into the hypolipidemic activity of various purified CPFP fractions. The results revealed a significant decrease in triglyceride (TG) content upon treatment with different CPFP purified fractions in comparison to the model group treated with 1 mM glucose (*p* < 0.05). Notably, CPFP-II exhibited the most prominent effect, followed by CPFP-III and CPFP-I. Moreover, treatment with different concentrations of CPFP-II and III demonstrated significant hypolipidemic effects compared to the model group (*p* < 0.05). Figure 4A indicates that, at a concentration of 250 µg/mL, the purified CPFP fractions (CPFP-I, II, III, and IV) decreased TG by 3.91%, 32.55%, 27.83%, and 1.65%, respectively. At higher concentrations of 500 µg/mL and 1000 µg/mL, the reductions in TG were more substantial (28.07%, 51.47%, 33.35%, and 1.91% for 500 µg/mL; 32.87%, 59.25%, 34.38%, and 9.55% for 1000 µg/mL) (Figure 4B,C). Notably, treatment with 1000 μg/mL CPFP-II fractions exhibited the most effective reduction in TG content (0.08 mmol/gprot vs. 0.20 mmol/gprot). This observation slightly contradicted the earlier result of bile-acid-binding capacity. Oil Red O staining was employed to analyze fat deposition in the nematodes. Figure 4A–C demonstrate that the model group exhibited larger and darker fat-stained areas compared to the control group, indicating the successful establishment of a high-fat *C. elegans* model. Treatment with CPFP-II and III led to lighter-colored fat-stained areas at various concentrations, which closely resembled the control group. This suggests that CPFP treatment reduced fat accumulation in the worms, with CPFP-II showing the most pronounced effects.

The phenolic compounds derived from the *C. polyodonta* flower exhibited varying substance compositions across different purified extraction fractions, and it is known that higher concentrations of bioactive compounds are more effective in demonstrating hypolipidemic abilities compared to lower concentrations [36]. Given the differences in substance composition among the four purified fractions, it is likely that fractions with a higher phenolic content are more efficient in reducing fat deposition. This observation aligns with the findings of Larissa et al. [37], who reported that the phenolic content of raw wild mango influenced its ability to lower blood glucose levels. In addition, phenolic compounds have been reported to reduce the lipid storage of *C. elegans* by promoting related pathways, which induces lipolysis. Yan et al. [38] indicated that luteolin reduces lipids in *C. elegans* by the central serotonin pathway. Consequently, the results suggest that CPFP-II and III, which contain varying substance compositions, possess strong in vivo hypolipidemic bioactivity. Hence, further exploration of the hypolipidemic mechanism of the higher-content individual substances from CPFP-II and III is warranted.

## 4. Conclusions

In this study, the isolation of bioactive compounds from the *C. polyodonta* flower, along with its antioxidant capacity and hypolipidemic activity, was investigated using different concentrations of aqueous ethanol with AB-8 macroporous resin chromatography. The flower extract was found to contain significant amounts of phenols, flavonoids, and proanthocyanidins, contributing to its high antioxidant capacity. CPFP-II demonstrated the highest extract yield and collected the majority of total phenolic content (TPC) and total flavonoid content (TFC), while CPFP-III primarily collected the majority of total proanthocyanidin content (TPAC). Among the fractions, CPFP-II consistently exhibited the highest antioxidant activity in DPPH, ABTS, and FRAP assays. Furthermore, in vivo and in vitro evaluations of purified fractions (CPFP-I, II, III, and IV) revealed significant hypolipidemic activity, with CPFP-II demonstrating the most promising effect. Notably, CPFP-II also showed strong binding to bile acids and effective inhibition of pancreatic lipase activity. These findings suggest that CPFP-II holds great potential for further exploration. This study sheds light on the composition and hypolipidemic properties of phenolic compounds from the *C. polyodonta* flower, paving the way for its potential application in functional foods and dietary supplements aimed at preventing hyperlipidemia. As with the majority of studies, the design of the current study is subject to limitations. Even though the hypolipidemic effect was studied in vivo and in vitro, further studies in vivo should also focus on the biological activity of these extracts in higher forms of animals (like mice) and in-depth mechanism research.

## Figures and Tables

**Table 1:** Total phenolic content (TPC), total flavonoid content (TFC), and total proanthocyanidins content (TPAC) of the different purified fractions (CPFP-I, CPFP-II, CPFP-III, CPFP-IV, and CPFP-V) and CPFP of the whole *C. polyodonta* flower.

	Mass of Dried Residue (mg)	TPC (mg GAE/g DW)	TFC (mg CE/g DW)	TPAC (mg PB2E/g DW)
CPFP-I	168.45 ± 35.00 ^c^	648.41 ± 5.72 ^c^	322.36 ± 6.23 ^c^	120.97 ± 11.14 ^c^
CPFP-II	994.50 ± 156.69 ^a^	909.27 ± 45.83 ^a^	658.36 ± 56.40 ^a^	136.81 ± 6.10 ^b^
CPFP-III	674.30 ± 117.38 ^b^	846.78 ± 17.83 ^b^	426.14 ± 15.85 ^b^	175.56 ± 4.16 ^a^
CPFP-IV	163.65 ± 6.01 ^c^	259.28 ± 4.04 ^d^	112.51 ± 4.59 ^d^	53.80 ± 1.70 ^d^
CPFP-V	148.45 ± 11.95 ^c^	17.43 ± 0.39 ^e^	7.49 ± 0.42 ^e^	4.49 ± 0.31 ^e^
CPFP	-	257.77 ± 8.50 ^d^	153.06 ± 20.08 ^d^	45.32 ± 0.45 ^d^

^a–e^ Data with different superscript lowercase letters in the same column are significantly different (*p* < 0.05); - means no data.

**Table 2:** The contents of fourteen phenolic compounds of CPFP and its different purified fractions (CPFP-I, CPFP-II, CPFP-III, CPFP-IV, and CPFP-V) in the whole *C. polyodonta* flower (mg/g DW).

Phenolic Compounds	CPFP-I	CPFP-II	CPFP-III	CPFP-IV	CPFP-V	CPFP
Gallic acid	3.03 ± 0.00 ^a^	1.63 ± 0.00 ^c^	2.16 ± 0.03 ^b^	-	-	0.29 ± 0.01 ^d^
Procyanidin B1	16.24 ± 0.32 ^a^	1.98 ± 0.03 ^b^	-	-	-	0.73 ± 0.04 ^c^
(+) catechin	81.35 ± 0.32 ^a^	21.19 ± 0.13 ^b^	-	-	-	6.39 ± 0.29 ^c^
Procyanidin B2	16.52 ± 0.12 ^b^	73.52 ± 4.92 ^a^	5.13 ± 0.05 ^c^	-	-	10.02 ± 0.45 ^bc^
Procyanidin B4	49.74 ± 0.79 ^b^	101.58 ± 1.67 ^a^	-	-	-	20.75 ± 0.87 ^c^
Epicatechin	37.58 ± 1.26 ^b^	143.26 ± 3.04 ^a^	4.08 ± 0.07 ^d^	-	-	26.51 ± 1.18 ^c^
Procyanidin C1	2.13 ± 0.03 ^d^	44.95 ± 1.20 ^a^	12.56 ± 0.63 ^b^	-	-	10.53 ± 0.48 ^c^
Rutin	-	-	3.10 ± 0.05 ^a^	0.59 ± 0.05 ^b^	-	0.37 ± 0.02 ^c^
Isoquercitrin	-	-	3.08 ± 0.08 ^a^	2.33 ± 0.10 ^b^	-	0.36 ± 0.02 ^c^
1,2,3,6-Tetragalloylglucose		15.64 ± 0.34 ^b^	43.69 ± 0.77 ^a^			7.97 ± 0.23 ^c^
Kaempferol-3-*O*-rutinoside	-	-	3.77 ± 0.18 ^b^	6.63 ± 0.20 ^a^	-	0.53 ± 0.03 ^c^
Astragaline	-	-	0.92 ± 0.01 ^b^	4.26 ± 0.17 ^a^	-	0.25 ± 0.02 ^c^
Quercitrin	-	-	4.49 ± 0.08 ^b^	16.06 ± 1.32 ^a^	-	1.13 ± 0.10 ^c^
Afzelin			0.21 ± 0.01 ^c^	36.21 ± 0.09 ^a^	-	1.32 ± 0.06 ^b^

^a–d^ Data with different superscript lowercase letters in the same row are significantly different (*p* < 0.05).

## Data Availability

The data set generated in this article can be provided to the corresponding author upon request.

## Supplementary Materials

The following supporting information can be downloaded at https://www.mdpi.com/article/10.3390/antiox13060662/s1. Figure S1: Effect of glucose concentration on triglyceride content and Oil red O staining in *C. elegans* N2. (^a–c^ Bars with different superscript lowercase letters are significantly different (*p* < 0.05)).
