# Supplementary material for: Purification of Phenolic Compounds from Camellia polyodonta Flower: Composition Analysis, Antioxidant Property, and Hypolipidemic Activity In Vitro and In Vivo
Source: Antioxidants (Basel). 2024 May 28;13(6):662. doi: 10.3390/antiox13060662 (PMC11200836; doi:10.3390/antiox13060662)
Supplement: Supplementary file 1 [file antioxidants-13-00662-s001.zip › antioxidants-2941843-supplementary.pdf]

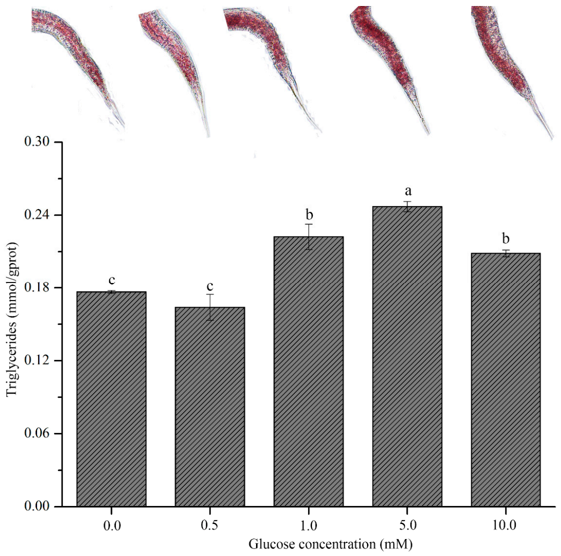

Supplementary Material Figure S1 Effect of glucose concentration on triglycerides content and Oil red O staining in *C. elegans* N2. ([a-c] Bar with different superscript lowercase letters were significantly different ($p < 0.05$))
